# Socioeconomic and lifestyle factors associated with HPV infection in pregnant women: a matched case-control study in Beijing, China

**DOI:** 10.1186/s12978-018-0645-x

**Published:** 2018-12-06

**Authors:** Xianhong Liang, Xianming Carroll, Wenyan Zhang, Wenjing Zhang, Gaifen Liu, Shangzhi Li, Sandra Leeper-Woodford

**Affiliations:** 1 Department of Neurology, Beijing Tiantan Hospital, Capital Medical University, Beijing, China; 2 China National Clinical Research Center for Neurological Diseases, Beijing, China; 3 Department of Public Health, Mercer University College of Health Professions, Atlanta, GA USA; 4 Department of Obstetrics and Gynecology, Beijing Chaoyang District Hospital of Maternal and Child Health, Beijing, China; 5 Department of Obstetrics, Beijing Chuiyangliu Hospital, Beijing, China; 6 Department of Biomedical Sciences, Mercer University School of Medicine, Macon, GA USA

**Keywords:** Human papillomavirus, Socioeconomic factor, Lifestyle factor, Alcohol consumption during pregnancy, Pregnant women

## Abstract

**Background:**

Human papillomavirus (HPV) infection plays key role in the development of cervical cancer. The purpose of this study was to investigate socioeconomic and lifestyle factors associated with HPV infection in pregnant women in Beijing, China.

**Methods:**

An age matched case-control study designed with 66 women as the case group (HPV positive) and 132 women as the control group (HPV negative) was carried out in two hospitals in Beijing. Socioeconomic and lifestyle factors were obtained using a standard questionnaire. Cervical cells from study subjects were collected for HPV detection. An unconditional logistic regression model with backward stepwise selection was performed to predict the odds ratio (OR) and 95% confidence interval (CI) for the significant factors associated with HPV infection.

**Results:**

The analyses of present data show that alcohol consumption during pregnancy was the strongest significant factor (OR = 3.35, 95% CI = 1.40–8.03, *p* = 0.007) when comparing the case (HPV positive) group with the control (HPV negative) group. There were no statistical differences observed in any of the socioeconomic factors when comparing the case and control groups.

**Conclusion:**

The results of this study may help to prevent HPV infection in China by providing evidence to support improving the national policy on alcohol restriction and introducing public health interventions, especially for pregnant women in Beijing.

## Plain English summary

Human papillomavirus (HPV) infection plays a key role in the development of cervical cancer. The prevalence of HPV infection is increasing in China, but the socioeconomic and lifestyle factors associated with HPV infection in pregnant Chinese women have not been systematically analyzed. In this age matched case-control study, we examined the effect of socioeconomic and lifestyle factors on 66 pregnant women (HPV positive) and 132 pregnant women (HPV negative) in two hospitals in Beijing. Our findings suggest that alcohol consumption during pregnancy is the strongest significant factor associated with HPV infection in Chinese women. There were no statistical differences observed in any of the socioeconomic factors when comparing the HPV positive and negative groups. Public health strategies that focus on regulation of the sales and consumption of alcohol in China, and development of health education programs for pregnant women, would be positive steps to approach solutions to this problem.

## Background

According to World Health Organization (WHO) statistical data, about 75,000 of the annual new cervical cancer cases were estimated to be in the People’s Republic of China, where cervical cancer was responsible for 33,000 deaths in 2008 [1]. Unlike other cancers with broad spectrum etiologies, cervical cancer is primarily caused by sexually transmitted human papillomavirus (HPV) infection [2–5]. The distribution and prevalence of HPV has been reported to vary by geographic region, and even among different areas in the same country [6, 7]. Although a series of studies have been performed to assess the prevalence of HPV genotypes in several regions in China, such as Yunnan Province [8], Zhejiang Province [9] and Guangdong Province [10], few studies have been reported in Beijing, the capital city in China.

Epidemiological studies show that higher prevalence rates of high-risk HPV infection and a higher proportion of women with multiple infections may be related to the age at first sexual intercourse [11], number of sexual partners and partner’s sexual behavior [12], multi-parity [13], and long-term use of oral contraceptives [14]. Other risk factors are still controversial including tobacco smoke [15], alcohol consumption, and socioeconomic factors such as education, occupation, and household income [16].

In the past two decades, China has witnessed rapid lifestyle and socioeconomic changes with increasing westernization, characterized by changes in behavior including exposure to tobacco smoke, increased alcohol consumption, changes in dietary choices, and physical inactivity [17]. Whether these changes in lifestyle patterns and other associated factors play roles in the increasing prevalence of HPV infection in China has not yet been investigated. The aim of this study was to investigate the relationship between socioeconomic/lifestyle factors and HPV infection among pregnant Chinese women in Beijing. The study hypotheses are: (1) when compared to pregnant women without HPV, pregnant women with HPV infection are more likely to be in the lower socioeconomic status (SES) as measured by education, occupation and household income; (2) when compared to pregnant women without HPV, pregnant women with HPV infection are more likely to choose unhealthy lifestyles as measured by tobacco smoking, alcohol consumption, and physical inactivity.

## Methods

### Study subjects

This study was designed as an age matched case-control study and conducted from January 2012 to June 2014. An HPV screening survey was used to test HPV status in the pregnant women. In this survey, 1684 pregnant women attending the two major prenatal care clinics, Beijing Chaoyang District Hospital of Maternal and Child Health and Beijing Chuiyangliu Hospital, were tested for cervical HPV-DNA status. After obtaining their consent, the pregnant women were tested for cervical HPV-DNA status at their obstetric visits (time course was 8–37 weeks of gestation) using the cervical specimen collection method, as described in the HPV detection section below.

Following the HPV screening survey at the initial visit, 68 pregnant women were tested as HPV positive (HPV prevalence 4.0%). Two of these women were excluded from the study because of missing birth dates, so that 66 pregnant women were identified as the case group (HPV positive), and 132 age-matched pregnant women were selected as the control group (HPV negative). The inclusion criteria for the HPV case and control groups included the absence of previous diagnoses of habitual miscarriage history, placenta previa, premature rupture of membranes, blood diseases and other complications. All 198 women signed a written informed consent and then completed a standardized questionnaire administered by personal interview. This standardized clinical form was also used to systematically collect all relevant clinical parameters during pregnancy [18]. Briefly this form included pregnancy-related information and other clinical parameters, as described in the section below: Biological factors and sexual behavior factors.

Ethics approval for this study was obtained from the Ethics Committee of Beijing Chaoyang District Hospital of Maternal and Child Health and Beijing Chuiyangliu Hospital. With respect to quality control in data collection, laboratory testing, and data entry, this clinical study was conducted in qualified medical laboratories by licensed medical doctors, nurses, and technicians. All procedures and methods were performed by the authors in accordance with the approved guidelines.

### HPV detection

Exfoliated cells in the cervical canal were collected from pregnant women by obstetricians using a specialized cervical brush. The cervical samples were kept in sample transport media for the cervical thin-prep cytologic test (TCT) and HPV detection, respectively. All samples were shipped to the lab at 4 °C and the tests were performed within 48 h. The TCT specimens were prepared, fixed and stained by a liquid-based cell sedimentation system. The GERVIST HPV genotype test kit was provided by the United States HOLOGIC company for the detection of a total of 14 sub-types of HPV, including high-risk HPV (16, 18, 31, 33, 35, 39, 45, 51, 52, 56, 58, 59, 66, 68). Each sample was examined to assess the quality of specimens, the number of cell types, and to include greater than 40% of the samples with cervical canal cells. A previous study [19] has suggested that detection rates of HPV with use of genotype tests were significantly higher than those of TCT, and that HPV detection is a better method for screening of cervical cancer.

### Assessments of socioeconomic factors

Socioeconomic status (SES) was assessed for education, occupation, and monthly household income. Three levels were assigned for education: low (≤ 9 years compulsory education), middle (9–12 years high school), high (> 12 years college and above). Occupation was separated into four different categories: housewife, manual labor, office worker, or other type of job. Monthly total household income was divided into four groups: < 3000 Chinese Yuan, 3000–5999 Chinese Yuan, 6000–8999 Chinese Yuan, or > 9000 Chinese Yuan (1.00 US Dollar ≈ 6.00 Chinese Yuan in 2014). In addition to these three well-known SES indicators, we measured the following factors which might also reflect social-demographic structures and social functions affecting the women’s health. These factors included belonging to either Chinese Han or a minority ethnic group, marital status including married or unmarried (single/divorced/widowed), length of residence in Beijing (> 5 years or ≤ 5 years), and living conditions (home owner, renter, living with parents, other facility).

### Assessment of lifestyle factors

Habitual smoking before pregnancy or during pregnancy was defined as continuously smoking one or more cigarettes per day for at least 6 months before pregnancy, or smoking one or more cigarettes per day during pregnancy. Environmental tobacco smoke (ETS) was defined as a non-smoker being exposed to another person’s tobacco smoke for at least 15 min daily for more than 1 day per week [20, 21]. Passive smoking at home from the husband, passive smoking from another family member, and passive smoking in the workplace were included as ETS variables. Alcohol consumption habits (consuming beer/wine/mixed alcohol drinks) were assessed using two questions: (1) Did you drink alcohol before the recognition of this pregnancy? (2) Did you drink alcohol at any stage after the recognition of this pregnancy? [22] The answers were divided into two groups: alcohol consumption (before or during pregnancy), or never drink. To report physical activity, the participants were asked the following question: During the last 30 days, on how many days were you physically active at least 60 min per day in a week? The answers were divided into the following: physical activity < 3 days in 1 week, or physical activity ≥ 3 days in 1 week.

### Biological factors and sexual behavior factors

The participants completed questionnaires that included pregnancy-related information, such as menarche age, length of menstrual cycle, age at first sexual intercourse, age at first pregnancy, number of previous pregnancies, number of live births, and number of abortions. The participants were also asked about their sexual behaviors such as how many sexual partners they had, how many sexual partners their husbands/life partners had, what kind of contraceptive methods they used, and if they and their husbands/life partners clean themselves before or after sexual intercourse. During the physical exam, the body mass index (BMI) of each woman was calculated as weight in kilogram divided by the square of body height in meters. Other findings, such as the presence of cervical erosion or cervicitis, vaginitis (trichomonas + mold), and TCT checks (trichomoniasis + mold) were also included as the covariates.

### Statistical analysis

Statistical analyses were performed using SAS, version 9.4 (SAS Institute Inc., Cary, NC, USA). In the first step, descriptive statistics were used to profile socioeconomic factors, lifestyle factors, biological factors and sexual behavior factors of all study subjects. HPV cases and controls were compared using student t-test for continuous variables and chi-square test for categorical variables [23]. The non-parametric test (Wilcoxon) test was used to analyze educational level and household income. To assess the association between all factors and HPV infection, multiple logistic regression analysis was performed with all independent variables simultaneously included in the same model [24].

The second step involved using the unconditional logistic regression analysis with backward stepwise procedures. This was performed based on the maximum partial likelihood estimates to construct the final best-fit model [25], and to identify the predictors of risk for HPV infection among all independent variables. This model retests those variables at *p* less than 0.2 to determine which variables have the strongest significance. The backward stepwise method [26] starts with all variables in the model, then removes the variable with the least statistically significant until all remaining variables have a significant *p*-value. We estimated odds ratio (OR) and 95% confidence interval (CI) for differing levels of exposure. All statistical tests were considered to be significant at an alpha level of 0.05 on a two-tailed test.

## Results

### Distribution of matched factors and socioeconomic factors in HPV cases and controls

As shown in Table 1, there was no statistical age difference between HPV case and control groups (mean age of 28.15 ± 4.38 in the case group, and mean age of 28.17 ± 4.33 in the control group). There was no statistical significance in any of the socioeconomic factors when comparing the HPV case and control groups (Table 1).

**Table 1:** Distribution of matched factor and socioeconomic factors in HPV infection: comparison of HPV cases and controls

Characteristics	HPV Cases *n* = 66 (%)	Controls *n* = 132 (%)	*p-value	OR (95%CI)	**p-value
Matched factor					
Age	28.15 ± 4.38	28.17 ± 4.33	0.977	–	–
Socioeconomic factor					
Ethic group			0.156		
Han nationality	64 (97.0)	121 (91.7)		1	
Other ethics	2 (3.0)	11 (8.3)		0.36(0.04–1.67)	0.278
Educational level			0.480		
Low(≤ 9 years)	11 (16.7)	35 (26.5)		0.65(0.27–1.57)	0.409
Middle (9–12 years)	27 (40.9)	41 (31.1)		1.23(0.61–2.50)	0.638
High(> 12 years)	28 (42.4)	56 (42.4)		1	
Occupation			0.687		
Housewife	28 (42.4)	63 (47.7)		1.06(0.34–3.55)	0.990
Manual labor	15 (22.7)	21 (15.9)		1.69(0.46–6.65)	0.552
Other type	16 (24.2)	32 (24.2)		1.18(0.34–4.43)	0.990
Office worker	7 (10.6)	16 (12.1)		1	
Household income			0.080		
< 3000 Yuan/month	20 (30.3)	28 (21.2)		1.16 (0.39–3.55)	0.972
3000–5999 Yuan/month	24 (36.4)	50 (37.9)		0.73 (0.27–2.06)	0.651
6000–8999 Yuan/month	10 (15.2)	34 (25.8)		0.46 (0.13–1.54)	0.249
≥ 9000 Yuan/month	12 (18.2)	20 (15.2)		1	
Marital status			0.475		
Married	64 (97.0)	130 (98.5)		1	
Single/Divorced/Widowed	2 (3.0)	2 (1.5)		2.0 (0.15–27.59)	0.815
Residency in Beijing			0.920		
≤ 5 years	34 (51.5)	67 (50.8)		1	
> 5 years	32 (48.5)	65 (49.2)		0.96 (0.47–1.97)	0.990
Living condition			0.269		
House owner	13 (19.7)	18 (13.6)		1	
Rental/Living with parents/Other	53 (80.3)	114 (86.4)		0.66 (0.29–1.54)	0.383

*p-value was obtained from student t-test for continuous variables and chi-square test for categorical variables. Non-parametric test (Wilcoxon) test for educational level and household income. **p-value was obtained from the multiple logistic regression model that simultaneously included socioeconomic factors

### Distribution of lifestyle factors in HPV cases and controls

In Table 2, descriptive analyses showed that HPV positive pregnant women were more likely to have lifestyle factors such as consumption of alcohol during pregnancy (*p* = 0.006), and exposure to passive smoking in the workplace (*p* = 0.037). In Table 2, multiple logistic regression analyses also showed a significant association between alcohol consumption during pregnancy and being HPV positive (OR = 3.53, 95% CI = 1.30–10.50, *p* = 0.011). However, passive smoking at the workplace and having HPV infection did not reach the significance level (*p* = 0.065).

**Table 2:** Distribution of lifestyle factors in HPV infection: comparison of HPV cases and controls

Characteristics	HPV Cases *n* = 66 (%)	Controls *n* = 132 (%)	*p-value	OR (95%CI)	**p-value
Lifestyle factor					
Tobacco smoking			0.885		
Smoking during pregnancy	9 (13.6)	19 (14.4)		0.94 (0.35–2.36)	0.999
Never	57 (86.4)	113 (85.6)		1	
Passive smoking at home from the husband			0.474		
Yes	29 (43.9)	51 (38.6)		1.24 (0.65–2.34)	0.577
No	37 (56.1)	81 (61.4)		1	
Passive smoking from other family member			0.380		
Yes	11 (16.7)	16 (12.1)		1.44 (0.57–3.54)	0.509
No	55 (83.3)	116 (87.9)		1	
Passive smoking in the workplace			0.037		
Yes	21 (32.3)	25 (18.9)		2.05 (0.96–4.50)	0.065
No	44 (67.7)	107 (81.1)		1	
Alcohol consumption before pregnancy			0.053		
Yes	20 (30.3)	24 (18.2)		1.93 (0.92–4.81)	0.132
No	46 (69.7)	108 (81.8)		1	
Alcohol consumption during pregnancy			0.006		
Yes	14 (21.2)	10 (7.6)		3.53 (1.30–10.50)	0.011
No	52 (78.8)	122 (92.4)		1	
Walking or biking 60 min			0.364		
< 3 days/week	15 (22.7)	38 (28.8)		0.74 (0.34–1.51)	0.477
≥ 3 days/week	51 (77.3)	94 (71.2)		1	

*p-value was obtained from student t-test for continuous variables and chi-square test for categorical variables. **p-value was obtained from the multiple logistic regression model that simultaneously included lifestyle factors

### Distribution of biological factors and sexual behavior factors in HPV cases and controls

The data in Table 3, show that there were no statistically significant associations observed in biological factors or sexual behavior factors in the HPV case and control groups.

**Table 3:** Distribution of biological factor and sexual behavior factors in HPV infection: comparison of HPV cases and controls

Characteristics	HPV Cases *n* = 66 (%)	Controls *n* = 132 (%)	*p-value	OR (95%CI)	**p-value
Biological factor					
Pre-pregnancy BMI kg/m^2^			0.739		
≥ 28	6 (9.1)	14 (10.6)		0.84 (0.25–2.49)	0.952
< 28	60 (90.9)	118 (89.4)		1	
Menarche age			0.379		
< 12 years old	1 (1.5)	127 (96.2)		0.35 (0.01–3.89)	0.658
≥ 12 years old	65 (98.5)	30 (22.7)		1	
Menstrual cycle (28 days)			0.211		
Abnormal	56 (84.8)	102 (77.3)		0.63 (0.26–1.40)	0.309
Normal	10 (15.2)	30 (22.7)		1	
Age at first sexual intercourse			0.685		
< 20 years old	5 (7.6)	8 (6.1)		1.28 (0.31–4.76)	0.896
≥ 20 years old	61 (92.4)	124 (93.9)		1	
Age at first pregnancy			0.169		
< 24 years old	27 (40.9)	41 (31.1)		1.74 (0.82–3.74)	0.165
≥ 24 years old	39 (59.1)	91 (68.9)		1	
Number of pregnancies			0.087		
≥ 2	30 (45.5)	77 (58.3)		0.59 (0.30–1.12)	0.113
0–1	36 (54.5)	55 (41.7)		1	
Number of parity			0.228		
≥ 1	38 (57.6)	64 (48.5)		1.54 (0.77–3.16)	0.251
0	28 (42.4)	68 (51.5)		1	
Number of abortions			0.477		
≥ 2	8 (12.1)	21 (15.9)		0.75 (0.28–1.82)	0.646
0–1	58 (87.9)	111 (84.1)		1	
Sexual behavior factor					
Number of sexual partners			0.065		
< 2	54 (81.8)	120 (90.9)		1	
≥ 2	12 (18.2)	12 (9.1)		2.6 (0.89–8.10)	0.084
Number of sexual partners of husband/life partner			0.433		
< 2	60 (90.9)	124 (93.9)		1	
≥ 2	6 (9.1)	8 (6.1)		1.67 (0.4–6.56)	0.578
Contraceptive methods			0.867		
Condom	37 (56.1)	76 (57.6)		1.05 (0.48–2.35)	0.990
Oral contraceptive + contraceptive ring	13 (19.7)	22 (16.7)		1.30 (0.44–3.82)	0.766
None	16 (24.2)	34 (25.8)		1	
Cleaning before sexual intercourse			0.440		
Neither /one person	17 (25.8)	41 (31.1)		0.77 (0.37–1.56)	0.546
Both	49 (74.2)	91 (68.9)		1	
Cervical erosion or cervicitis			0.427		
No	46 (69.7)	99 (75.0)		1	
Yes	20 (30.3)	33 (25.0)		1.32 (0.63–2.75)	0.519
Vaginitis (trichomonas + mold)			0.449		
No	56 (84.8)	117 (88.6)		1	
Yes	10 (15.2)	15 (11.4)		1.39 (0.52–3.60)	0.589
TCT check (trichomoniasis + mold)			0.569		
No	60 (90.9)	123 (93.2)		1	
Yes	6 (9.1)	9 (6.8)		1.40 (0.37–5.02)	0.753

*p-value was obtained from student t-test for continuous variables and chi-square test for categorical variables. **p-value was obtained from the multiple logistic regression model that simultaneously included biological factor and sexual behavior factors

### Best-fit model predicting the strongest association between all factors and HPV infection

In the final best-fit model (Table 4), only one independent variable, alcohol consumption during pregnancy, remained in the best-fit model showing association with HPV infection (OR = 3.35, 95% CI = 1.40–8.03, *p* = 0.007). This significant result was unchanged from estimates of association observed in the original regression model (Table 2). Other independent variables due to non-significant results were consequences removed from the final best-fit model (Table 4).

**Table 4:** Associated factors identified in backward stepwise logistic regression (best-fit) Model

Variable	*Estimate	Standard Error	Wald Test	*p*-value	**OR (95%CI)
Alcohol consumption during pregnancy	1.21	0.45	7.33	0.007	3.35 (1.40–8.03)

*Values are the estimated non-standardized regression coefficients. **OR indicates likelihood of HPV infection. Variables entered into the Model: Ethic group, household income, passive smoking at workplace, alcohol consumption before pregnancy, alcohol consumption during pregnancy, number of sexual partners, age at first pregnancy, number of pregnancies, cervical erosion or cervicitis, vaginitis (trichomonas + mold), and TCT check (trichomoniasis + mold)

## Discussion

In this study, we examined the effects of socioeconomic and lifestyle factors associated with HPV infection in an age matched case-control study among pregnant women in Beijing. The results showed that, compared to HPV negative women, HPV positive women were more likely to be associated with the lifestyle factor of consuming alcohol during pregnancy (Table 4).

For a number of reasons, alcohol consumption in pregnancy is an especially important factor to advise pregnant women to avoid [27, 28]. Alcohol may be a potent modulator of immune function which can lead to immune deficiency and increased susceptibility to various chronic and infectious diseases [27, 28]. Not only chronic alcohol abuse but also acute and moderate alcohol consumption can adversely affect the immune system [27]. The normal defense responses to various pathogens are divided into two phases: the first phase is an inflammatory reaction, which provides protection against the immediate effects of the infection, and the second phase involves the development of immunity to the pathogen. Alcohol consumption is known to interfere with both of these phases in the normal immune response [27].

With respect to socializing in China, alcohol is quite a powerful factor for those involved in social interactions and business deals, and there has been a steady increase in alcohol production and consumption and in rates of alcohol-related conditions in China [29]. These dramatic increases were noted after the 1980s and stem from China’s fast economic development and the parallel rise in average income level [30]. Drinking alcoholic beverages has been traditionally accepted in China during major social events, such as the spring festival, wedding ceremonies and birthday parties. However, the rapid growth in the Chinese economy has been accompanied by noticeable changes in the drinking behavior of the Chinese population. Alcohol is now commonly consumed to relieve stress and foster good relations between supervisors and employees, while dining together after work or during business meetings over dinner [29, 30]. Beijing Chaoyang district where our present survey was conducted is a famous business district with many white-collar workers living and working there, including women who frequently consume alcohol during their working hours and social activities.

The percent of women consuming alcohol during pregnancy has been reported as a serious problem in other countries, such as 32.5% in Congo [31], 4.4% in India [32]. A study in Korea [33] suggests that women who drink alcohol may have an increased risk of persistent HPV. They tested 9230 women for HPV and found that current drinkers were nearly three times more likely than non-drinkers to test positive for HPV at the beginning of the study, and then again at a 2-year follow-up.

Currently, there are few published data on the association between alcohol consumption and HPV infection among pregnant women in China. In our study, we found that women reporting alcohol consumption during pregnancy 3.5 times more likely to be associated with HPV infection than those who never drank (Table 4). Serious side effects related to alcohol consumption during pregnancy had been previously reported even with light drinking, which led to a recommendation by the world health organization (WHO) of “no alcohol at all during pregnancy”.

China urgently needs to develop a comprehensive national alcohol control policy based on the experience of other countries and on WHO recommendations. This may be done by adopting measures aimed at controlling overall alcohol consumption (a population-based approach) as well as measures intended to reduce risky behavior (a high-risk approach). It is also likely that restrictions on alcohol advertisements, taxation of alcoholic beverages, and establishing a legal age for drinking will have a significant impact on the frequency of many of these alcohol-related problems.

Based on this study’s results, we would suggest that pregnant women who consume alcohol as a special target group for educational and prevention measures. These women may not know the impact of alcohol consumption on their health, and should be educated on how their risky behavior choices may affect them and their developing fetus. Therefore, we recommend specific health education campaigns focused on pregnant women, especially for those who live or work in the Chaoyang District in Beijing.

Tobacco smoking has been classified as a possible risk factor of cervical cancer, but the effect of smoking on the risk of HPV infection is unclear [34]. In China, while the cigarette smoking rate has been very low in women, it has been very high in men, and this puts women at a high risk of problems due to passive smoking. A pooled analysis from a series of case studies indicated that passive smoking is a potential risk factor associated with invasive cervical cancer [35]. In contrast to these findings, the absence of significant associations between passive smoking and HPV infection was unexpected in our study. The reasons may due to the interaction between the related independent variables such as alcohol consumption during pregnancy and passive smoking at the workplace (correlation: *r* = 0.16, *p* = 0.024) that could influence the impact of passive smoking in the final best fit model analyses. Further investigations are needed before definitive conclusions can be made about the possible effects of this passive smoking factor on HPV infection.

In our current study, we found no relationship between socioeconomic indicators (education, occupation, and household income) and HPV infection (Table 1). The absence of this association may be due to the small sample size of this study which could affect the statistically significant of socioeconomic factors on HPV infection. A previous study in Sweden found that lower socioeconomic status was associated with HPV vaccination status [36]. In China, women have been vaccinated against HPV in the last 2 years [37], but the relationship between socioeconomic factors and HPV vaccination status remain unknown, and perhaps should be explored in future studies.

Certain limitations should be considered in interpreting the results of the current study. First, this is a hospital-based case-control study. Due to the mutual selection relationship between pregnant women and hospitals, the representative of the research subjects was limited, which had a certain impact on the research results. Second, the sample size of this study is very small and the prevalence of HPV positive status is relatively low (4.0%) in pregnant Chinese women. These factors may limit the statistical power for detecting a significant difference in our studies. Third, because of the limited time of this study period, we did not identify the genotypes of those HPV infections. Future studies may be needed to investigate this further. Fourth, case-control studies may only offer hints as to the causative factors that may lead to the association of HPV infection, and the use of terms such as “prediction” and “risk” do not imply causal or temporal relationships. Lastly, retrospective self-reporting of behavioral risk factors such as number of sexual partners may be susceptible to under-reporting due to culture differences and lack of recall. The variables such as tobacco and alcohol exposure may also be susceptible to recall bias. Future studies may be proposed to address these limitations.

## Conclusion

In summary, these data suggest that in these pregnant Chinese women, HPV infection may be associated with alcohol consumption during pregnancy. HPV infection may be prevented through public health intervention such as HPV vaccinations and lifestyle modifications, but the adoption of a healthy lifestyle, such as not consuming alcohol in pregnancy, requires not only individual behavioral changes but also changes in the social environment. Public health strategies that focus on regulation of the sales and consumption of alcohol in China, and development of health education programs, especially for pregnant women in Beijing, would be positive steps to approaching solutions to these health problems.

## Abbreviations

BMI: Body mass index
CI: Confidence interval
ETS: Environmental tobacco smoke
HPV: Human papillomavirus
OR: Odds ratio
SES: Socioeconomic status
TCT: Thin-prep cytologic test
WHO: World Health Organization
